# NMR-Guided Repositioning of Non-Steroidal Anti-Inflammatory Drugs into Tight Junction Modulators

**DOI:** 10.3390/ijms22052583

**Published:** 2021-03-04

**Authors:** Takeshi Tenno, Kohki Kataoka, Natsuko Goda, Hidekazu Hiroaki

**Affiliations:** 1 Laboratory of Structural and Molecular Pharmacology, Graduate School of Pharmaceutical Sciences, Nagoya University, Nagoya 464-8601, Aichi, Japan; tenno.takeshi@e.mbox.nagoya-u.ac.jp (T.T.); tenno.natsuko@f.mbox.nagoya-u.ac.jp (N.G.); 2 BeCellBar, LLC, Nagoya 464-8601, Aichi, Japan; 3 Department of Biological Sciences, Faculty of Science, Nagoya University, Nagoya 464-8602, Aichi, Japan; kataoka.koki@c.mbox.nagoya-u.ac.jp

**Keywords:** dynamic equilibrium of tight junction, drug absorption enhancer, drug repositioning, hierarchical clustering, NMR screening, non-steroidal anti-inflammatory drugs, tight junction integrity

## Abstract

Bioavailability is a major bottleneck in the clinical application of medium molecular weight therapeutics, including protein and peptide drugs. Paracellular transport of these molecules is hampered by intercellular tight junction (TJ) complexes. Therefore, safe chemical regulators for TJ loosening are desired. Here, we showed a potential application of select non-steroidal anti-inflammatory drugs (NSAIDs) as TJ modulators. Based on our previous observation that diclofenac and flufenamic acid directly bound various PDZ domains with a broad specificity, we applied solution nuclear magnetic resonance techniques to examine the interaction of other NSAIDs and the first PDZ domain (PDZ1) of zonula occludens (ZO)-1, ZO-1(PDZ1). Inhibition of ZO-1(PDZ1) is expected to provide loosening of the epithelial barrier function because the domain plays a crucial role in maintaining TJ integrity. Accordingly, diclofenac and indomethacin were found to decrease the subcellular localization of claudin (CLD)-2 but not occludin and ZO-1 at the apicolateral intercellular compartment of Madin–Darby canine kidney (MDCK) II cells. These NSAIDs exhibited 125–155% improved paracellular efflux of fluorescein isothiocyanate insulin for the Caco-2 cell monolayer. We propose that these NSAIDs can be repurposed as drug absorption enhancers for peptide drugs.

## 1. Introduction

Peptide and protein therapeutics, including non-natural cyclic peptides, are an emerging class of pharmaceuticals in drug discovery for unmet medical needs [1,2,3]. They are characterized by a large molecular surface with different functional groups that can provide higher affinity and specificity to the therapeutic target proteins compared to the authentic “rule-of-five” (Ro5) drugs. However, such “beyond-Ro5 drugs” have a critical drawback, which is their poor bioavailability. Peptide and protein therapeutics can merely penetrate the epithelial barrier to reach target organs. Paracellular transport across the epithelial cell monolayer is inefficient for molecules with a molecular weight of over 500. Therefore, most peptide and protein therapeutics and other beyond-Ro5 drugs are only administrated via injection. Consequently, there is an urgent need to address the development of oral, buccal, nasal, and/or transdermal methods of administration for beyond-Ro5 drugs [4,5,6]. 

**Figure 1:**
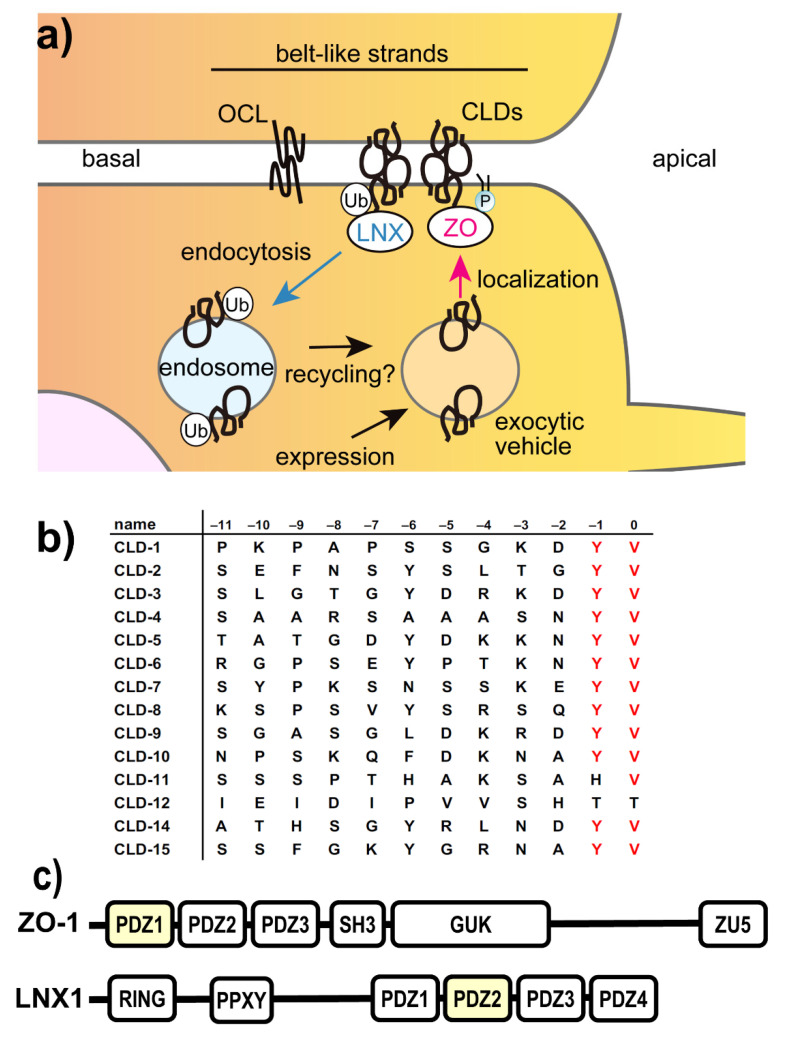
Components of tight junction complexes and the key molecular interactions among regulatory proteins and claudins. (**a**) Schematic overview of dynamic equilibrium between biogenesis and internalization of tight junction (TJ) components, claudins (CLDs), and occludin (OCL), associated with either ZO-1/2/3 (ZO) proteins or ligand-of-NUMB X1 (LNX). P denotes phosphatidyl-inositol phosphates, and Ub denotes ubiquitin. (**b**) Amino acid sequences of PDZ-binding motifs at the C-termini of human CLDs. The conserved C-terminal residues are colored in red. (**c**) Domain architectures of ZO-1 and LNX1 proteins. RING; RING domain, PDZ, PDZ domain; PPXY, NUMB binding motif; SH3, Src homology 3 domain; GUK, guanylate kinase domain; ZU5, ZO-1, and Unc5 homology domain. PDZ domains responsible for CLD interaction are colored yellow.

Tight junctions (TJs) are intercellular adhesion machinery located at the apical-most space of polarized epithelial cells, which are composed of the transmembrane proteins claudins (CLDs) and occludin (OCL) [7,8]. CLDs and OCL are associated with several intracellular scaffold proteins, including zonula occludens (ZO)-1/2/3 proteins and ligand-of-NUMB X1 (LNX1) protein (Figure 1a) [9]. The former scaffolding proteins connect the membrane proteins of TJs to actin cytoskeletons. TJs play a pivotal role in the barrier function of the paracellular transport pathway. Accordingly, chemical modifiers of TJ integrity are expected to act as drug absorption enhancers for beyond-Ro5 drugs.

To rationally discover the TJ-mitigating compounds, we focused on the protein-protein interaction of ZO-1 and CLDs. It has been shown that ZO-1 (and its close homolog ZO-2) is indispensable for the biogenesis of TJs and the maintenance of TJ integrity [10,11,12]. ZO-1/2 harbors three consecutive PSD-95/Discs large/and ZO-1 (PDZ) domains at the N-terminus of the protein. They follow a Src homology domain, the guanylate kinase (GUK) domain, and a long intrinsically disordered region that contains the actin-binding site (Figure 1c). At the same time, most (15 among 21) CLDs harbor a canonical PDZ domain-binding motif (PBM) at their C-termini. A PBM is characterized by two to four consecutive hydrophobic/aromatic residues. For CLDs, -*XX*YV-COOH, where *X* is any amino acid, is the conserved key motif (Figure 1b). This motif is recognized by the first PDZ domain of ZO-1 (ZO-1(PDZ1)), originally shown by the domain-deletion mutant experiments [12], and this specific interaction with CLDs seems necessary for TJ formation. According to the literature, an exogenous expression of the engineered ZO-1 fragment, which harbors the first PDZ domain followed by an artificial dimerization domain, is sufficient to form CLD belt-like assembly on the cell–cell junction, similar to that of TJs [12]. The claudin–ZO-2(PDZ1) interaction was also shown to independently control TJ formation [11]. A combination of gene knock-out/knock-down experiments with ZO proteins resulted in the severe malformation of TJ structure with poor barrier function [13]. The structural basis of this critical interaction was further studied by X-ray crystallography [14]. In the study, the four most C-terminal residues of CLDs were shown to essentially contact the PDZ domain.

In our previous study, we showed that the domain ZO-1(PDZ1) could weakly bind two different categories of small molecules, the head group of phosphatidylinositol phosphates [15] and baicalin/baicalein [16], at the same canonical CLD-binding pocket. We also showed that potential molecular competition between phosphatidylinositol phosphate and CLDs in ZO-1 binding may regulate TJ integrity [15]. In addition, either baicalin or baicalein could reduce the barrier function of the Madin–Darby canine kidney (MDCK) II cell monolayer by ZO-1(PDZ1) inhibition in combination with the activation of the TGFβ signaling pathway [16]. 

**Figure 2:**
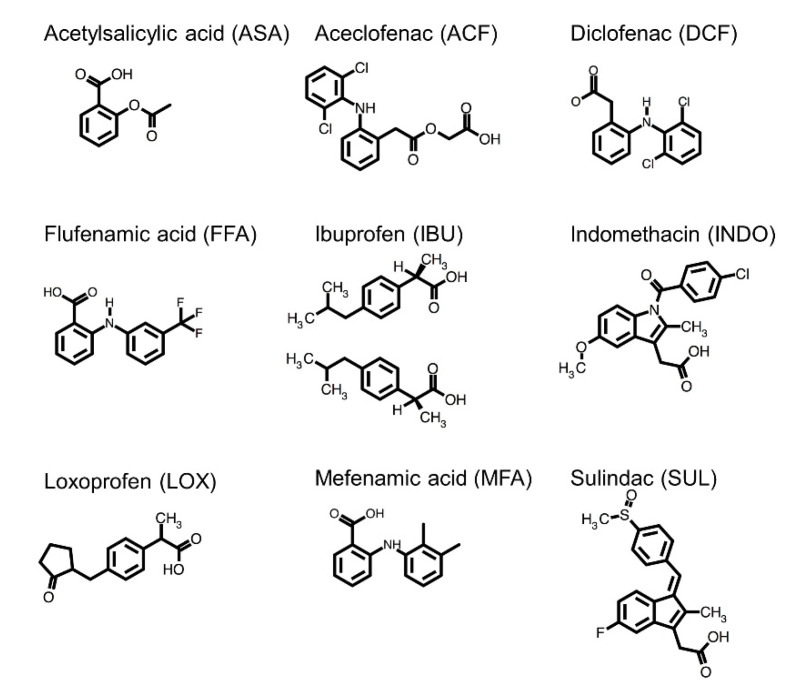
Chemical structures of non-steroidal anti-inflammatory drugs (NSAIDs) and their abbreviations: acetylsalicylic acid (ASA), aceclofenac (ACF), diclofenac (DCF), flufenamic acid (FFA), ibuprofen (IBU), indomethacin (INDO), loxoprofen (LOX), mefenamic acid (MFA), and sulindac (SUL).

Based on our previous observation, we chose several non-steroidal anti-inflammatory drugs (NSAIDs) as candidate molecules for screening ZO-1(PDZ1) inhibitors (Figure 2). We previously demonstrated that two NSAIDs, flufenamic acid and diclofenac, bound several PDZ domains with a broad specificity [17]. Among the examined PDZ domains, we observed a direct interaction between ZO-1(PDZ1) and either flufenamic acid or diclofenac with an estimated *K_d_* of 0.7 mM and 1.4 mM, respectively. Both compounds possess a carboxylic group, and we interpreted that this carboxylic group was the determinant of PDZ domain binding. It should be noted that recognition of the C-terminal -COOH group of PBM is the essential molecular function of PDZ domains [18]. However, it is noted that most NSAIDs harbor a carboxylic group. NSAIDs competitively inhibit either cyclooxygenase (COX)-1, COX-2, or both, and the substrate of these enzymes is arachidonic acid [19,20]. Its carboxylic group is critical for COX binding. Therefore, we hypothesized that the other NSAIDs can bind to ZO-1(PDZ1) and inhibit the interaction between the ZO-1 and CLDs. This hypothesis is a conceptual extension of drug profile matching (DPM) in which a virtual affinity fingerprint of many compounds against a single drug target can be used to predict new candidate molecules for the other drug target with a similar affinity fingerprint [21].

In this study, we examined the direct interaction between ZO-1(PDZ1) and the nine NSAIDs listed in Figure 2 using the solution nuclear magnetic resonance (NMR) technique. Five had weak but specific binding to the canonical binding site of ZO-1(PDZ1), indicating our expanded DPM approach is efficient. We selected some NSAIDs and subjected them to further pharmacological study of TJ integrity using MDCK II cells and the barrier function in Caco-2 cells. The TJ opening activity for diclofenac and indomethacin were observed.

## 2. Results

### 2.1. Direct Interaction of Several NSAIDs with ZO-1(PDZ1) Revealed by NMR Titration Experiment

NMR titration experiments (formally called chemical shift perturbation (CSP) mapping experiments) represent an excellent method of examining the direct interaction between a protein and its ligands. The method has at least three advantages over the other analytical solution methods: (1) sensitivity in weak interaction, (2) spatial resolution of interaction surfaces if the target protein signals are fully assigned, and (3) discriminability between specific and non-specific interaction. It should be noted that the last issue, discrimination between specific and non-specific interaction, may become difficult in some cases because CSPs are also influenced by an allosteric effect of the ligand binding. In such a case, transferred cross-saturation experiments should be supplementarily used [22]. Although certain demerits exist, NMR titration experiments are attractive at the early stage of drug discovery. We already reported the weak direct interaction between ZO-1(PDZ1) and either diclofenac (DCF) or flufenamic acid (FFA), deduced by the NMR titration experiments of fine data points [17]. In addition, since there was literature that said another NSAID, sulindac (SUL), could directly bind and inhibit the PDZ domain of Dishevelled, we hypothesized that SUL can also bind ZO-1(PDZ1) [23]. Including these three NSAIDs, we systematically compared the nine NSAIDs listed in Figure 2 in terms of their binding to ZO-1(PDZ1) using NMR titration experiments.

**Figure 3:**
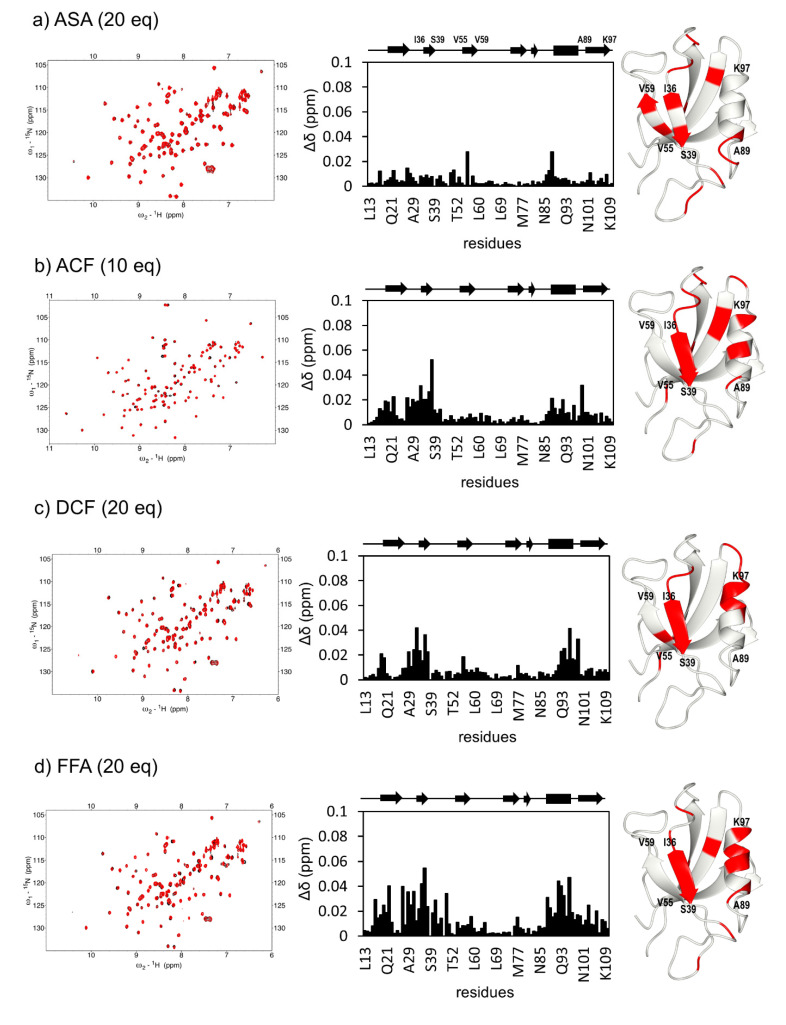

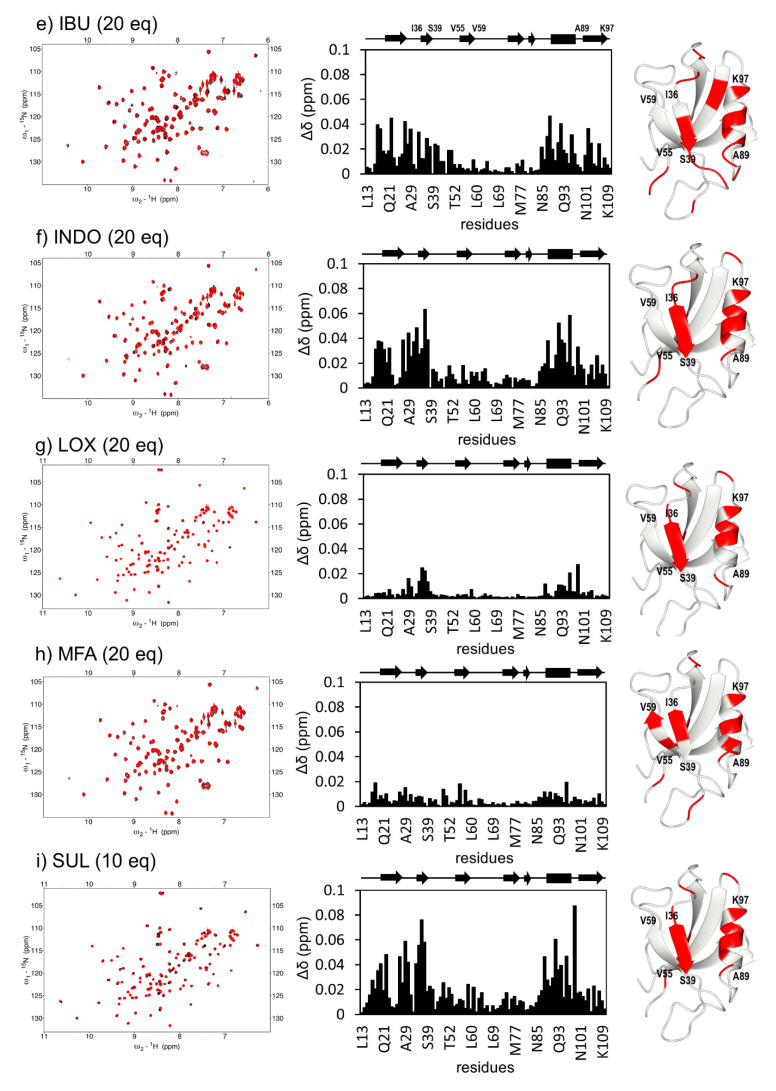
Interaction between ZO-1(PDZ1) and the NSAIDs. Overlaid heteronuclear single quantum coherence (HSQC) spectra of 0.1 mM ZO-1(PDZ1) in the absence (black) and presence of NSAIDs of the indicated molar equivalent (left), the combined chemical shift changes plotted versus the residues of ZO-1(PDZ1) (middle), and resonances representing residues with larger chemical shift changes than the threshold values are mapped onto the ribbon model of mouse ZO-1(PDZ1) (PDB: 2H3M) (right). (**a**) ASA (20 eq, 2.0 mM); (**b**) ACF (10 eq, 1.0 mM); (**c**) DCF (20 eq); (**d**) FFA (20 eq); (**e**) IBU (20 eq); (**f**) INDO (20 eq); (**g**) LOX (20 eq), (**h**) MFA (20 eq), and (**i**) SUL (10 eq).

We found a weak interaction between ZO-1(PDZ1) and five NSAIDs, aceclofenac (ACF), DCF, FFA, indomethacin (INDO), and SUL, using superimposed ^1^H-^15^N two-dimensional spectra (Figure 3b–d,f,i, left panels, respectively). Among them, we confirmed a direct interaction between DCF and ZO-1(PDZ1) by the mutation experiments [17]. Based on the similar tendency of the residues showing CSPs, we assumed the other four NSAIDs also bound ZO-1(PDZ1) directly. The combined chemical shift changes were calculated and plotted against the residues (middle panels) [24]. Changes larger than the average + 1 sigma were mapped onto the solution structure of ZO-1(PDZ1) (PDB: 2H3M; Figure 3, right panels) and visually inspected. In contrast, the addition of either acetylsalicylic acid (ASA) or mefenamic acid (MFA) showed a different pattern of CSPs with relatively weak signal changes although several residues were common with those of the other “strong” NSAIDs (Figure 3a,h, respectively). In this study, we temporarily classified that ASA and MFA did not bind ZO-1(PDZ1). Accordingly, in the case of ibuprofen (IBU) and loxoprofen (LOX) titration, certain CSPs were also observed, but the appearance of the residue sets showing large CSP values was not similar to the previous pattern of “strong” NSAIDs cases. For example, although CSPs were small LOX induced characteristic two clusters of CSPs around residue 35 (2) and 100 (2), whereas IBU induced larger CSPs upon broader range of residues from 18 to 48. In theory, the results of the NMR titration experiment with the protein can be adopted to the two-state transition between the free state and the ligand-bound state if a sufficient amount of the ligand was added to the target protein. However, if the affinity of the ligand to the target protein is not high enough, discriminating between non-specific and specific binding becomes difficult. To analyze these CSP results, we employed a hierarchical cluster analysis.

### 2.2. Analysis of CSP Data Using Hierarchical Clustering

In our previous study, we proposed the use of principal component analysis (PCA) instead of CSP mapping [25]. The use of PCA to analyze a series of NMR titration experiments was initially introduced by Sakurai et al., who showed the advantages of the method when studying the folding intermediate of βMG [26,27]. PCA is effective in visualizing the similarities and differences in heteronuclear single quantum coherence (HSQC) chemical shift information about the reduced dimensions, such as 2D or 3D. However, the method does not provide cluster analysis and classification of the ligands based on their CSP data. In this study, we employed a hierarchical cluster analysis instead of a PCA and intended to classify NSAIDs based on their different CSPs in ZO-1(PDZ1) binding (Figure 3). In theory, CSP data may contain two distinct pieces of information, affinity and contact residues. Since most NSAIDs can bind ZO-1(PDZ1) weakly, we did not intend to compare their binding affinity at this time but intended to differentiate various contact residues, which may indicate the binding modes of each NSAID. We, therefore, decided to use the cosine similarity of CSP data for clustering instead of Euclidean distances. Note that we used the combined chemical shift changes for the ^1^H and ^15^N axis, whereas ^1^H-CSP and ^15^N-CSP were analyzed separately in our previous PCA analysis for Aβ(1-42). Again, this is because our purpose is to classify ligands by their different contact residues. In addition, since we employed cosine similarity, only the CSP data of a fixed drug concentration were necessary for the analysis.

**Figure 4:**
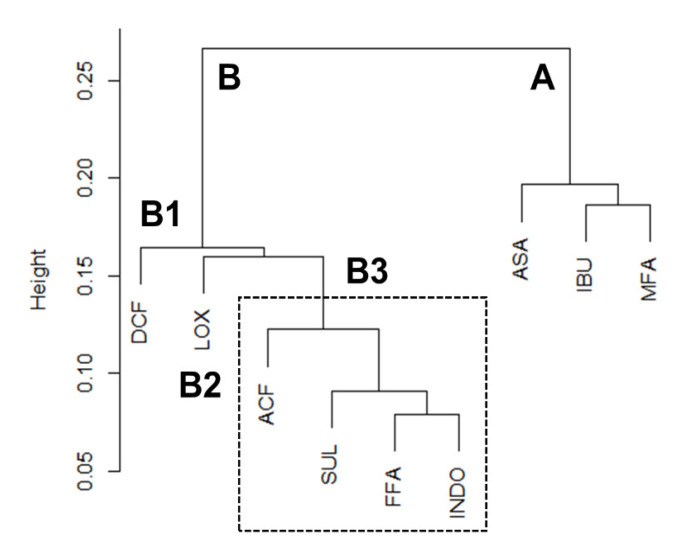
Cluster analysis of nuclear magnetic resonance (NMR). Combined chemical shift changes per each residue were vectorized and analyzed by hierarchical cluster analysis. NSAIDs were divided into two clusters (A and B), specific binders and non-specific binders, respectively. The cluster B is further divided in three (B1, B2, andB3) clusters (see text).

After hierarchical cluster analysis, the nine NSAIDs were classified into two clusters, the first one (Figure 4, cluster A, right) contains ASA, IBU, and MFA, and the second cluster (cluster B, left) contains DCF, LOX, ACF, SUL, FFA, and INDO. Since we already identified that DCF specifically bound to the canonical ligand-binding site of ZO-1(PDZ1), cluster B is assumed to be the NSAIDs that binds ZO-1(PDZ1) specifically. On the other hand, we tentatively interpreted that cluster A is reflected by non-specific ligand-protein interaction. Note that IBU showed a certain CSP when titrated with 20 molar equivalents. However, the exhibited CSP pattern seemed rather non-specific, despite the relative amount of signal changes. This is a remarkable feature of the hierarchical clustering approach for CSP data. Cluster B consists of six NSAIDs that specifically bind ZO-1(PDZ1). These NSAIDs occupy the canonical peptide-binding pocket between α1 and β2. This cluster is subdivided into three subclusters, B1, B2, and B3. Cluster B1 contains DCF, cluster B2 contains LOX, and cluster B3 contains ACF, SUL, FFA, and INDO (Figure 4). Although the absolute value of the residue-averaged CSP for LOX is rather small, LOX is considered to be a specific ZO-1(PDZ1) ligand. Notably, SUL and INDO belong to the same sub-cluster (B3), and their chemical structures are also very similar. We assumed they have a remarkably similar binding pose to ZO-1(PDZ1). However, notably, the other NSAIDs belong to different subclusters, although their chemical structures are similar. For example, FFA specifically bound to ZO-1(PDZ1), whereas MFA did not. These observations partly support the idea that even only a small modification of the NSAIDs’ chemical structure may alter the binding pose against the pocket of ZO-1(PDZ1), which was sensitively indicated by CSPs.

### 2.3. TJ Reduction Activity of DCF, FFA, and INDO

**Figure 5:**
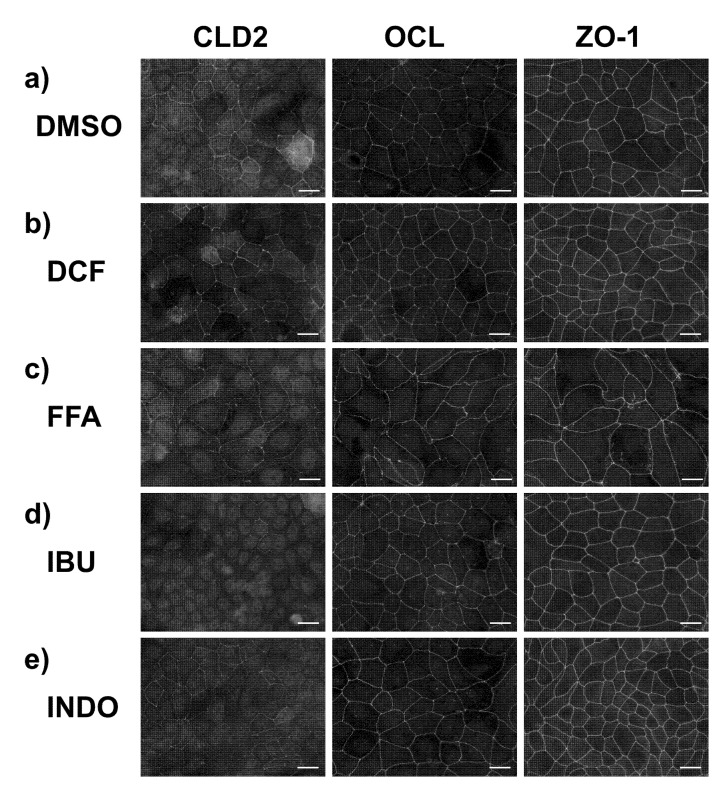
Effects of NSAIDs on tight junction integrity of MDCK II cells. Immunofluorescence staining of claudin-2 (CLD-2, left), occludin (OCL, middle), and ZO-1 (right) images are arrayed. Cells were treated with NSAIDs at a concentration of 100 μM for 48 h. (**a**) control (DMSO), (**b**) DCF, (**c**) FFA, (**d**) IBU, and (**e**) INDO. Scale bar = 20 µM. Brightness is modified to 160%.

We subsequently examined the TJ modulating activity of the four NSAIDs—DCF, FFA, IBU, and INDO—using MDCK II cells, as this cell system has been extensively studied by TJ researchers. We selected CLD-2 and OCL as the markers for TJ integrity. We also chose ZO-1 as the representative TJ scaffold protein. Note that CLD-2 is one of the most abundant CLDs in MDCK II cells [28]. After exposure of the 100 µM NSAIDs, CLD-2—most of which localized at the apicolateral cell–cell junctional compartment with the remaining molecules found in the cytosol—changed its subcellular localization. In the case of DCF, FFA, and INDO, CLD-2 localized at the cell–cell junction virtually disappeared. Although some CLD-2 were partly visible at the junction, only the internalized portion was visible (Figure 5). For all NSAID-treated cells, the subcellular localization of OCL and ZO-1 did not change. Therefore, the three NSAIDs, DCF, FFA, and INDO, only affected CLD-2 in this study. Consequently, we assumed that the fundamental architecture of TJs was maintained and not severely damaged by these NSAIDs at the examined concentration (100 µM). In addition, we found an unexpected change in the size and area of the cells treated by FFA (Figure 5c, middle and right panels). We already observed morphological changes of MDCK II cells from a cobblestone-like shape to a slender fibroblast-like cell shape when treated with a high dose of baicalein and quercetin [16,29]. This case was not similar to the previous observation because the shape of FFA-treated cells was not slender but expanded.

### 2.4. Analysis of the Barrier Function of TJs of the Caco-2 Cell Monolayer Treated by DCF and INDO

**Figure 6:**
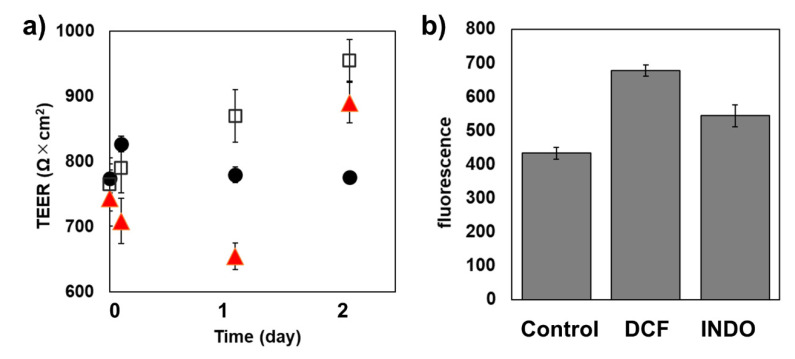
Effects of DCF and INDO on transepithelial electrical resistance (TEER) changes and paracellular efflux of fluorescence-labeled insulin of Caco-2 cell monolayers. (**a**) TEER changes. Filled circle, control (0.3% DMSO); red triangle, 300 μM DCF; open square, 300 μM INDO, (**b**) paracellular efflux of FITC-insulin. Cells were treated with NSAIDs (DCF or INDO) at a concentration of 300 μM for 48 h. The cells treated with 0.3% DMSO were set as a control. Error bars indicate standard deviations.

Of the NSAIDs examined above, we selected DCF and INDO to subject to the functional analysis of the TJ barrier using the Caco-2 cell monolayer, a common in vitro model for the intestinal epithelial barrier. Caco-2 cells were cultured to form a tight monolayer on the upper filter of the transwell filter plate and then subjected to treatment by 300 µM of either DCF or INDO. First, we examined the time-dependent change of the transepithelial electrical resistance (TEER) of the Caco-2 cell monolayers at 1 h, 1 day, and 2 days (Figure 6a). When the Caco-2 cells were exposed to DCF, TEER slightly decreased at 1 h and decreased further (~18%) at 1 day. TEER recovered to 120% after 2 days. The cells treated by INDO exhibited a mild increase in TEER after drug treatment. These results were not fully consistent with our previous microscopic observation that the subcellular localization of CLD-2 at the TJ compartment decreased drastically in MDCK II.

Next, we examined the change in the transepithelial flux of the fluorescein isothiocyanate (FITC)-labeled insulin, the molecular weight of which was approximately 6000. To demonstrate the activity of the drug permeability enhancement of DCF and INDO, the paracellular permeability of insulin over the Caco-2 cell monolayer as an intestinal mucosal barrier model was examined (Figure 6b). After 24 h of treatment, DCF and INDO showed +55% and +25% permeability enhancement of insulin, respectively. DCF showed more significant flux enhancement. This was somewhat consistent in that DCF but not INDO showed a decrease in TEER. Finally, the results showed the potential of DCF and INDO as a drug absorption enhancer in the development of the oral administration of insulin for diabetes patients.

## 3. Discussion

In this study, we employed a drug repositioning approach for NSAIDs and aimed to demonstrate the potential use of the selected NSAIDs as drug absorption enhancers. As a result, DCF and INDO were found to be candidates. Since these NSAIDs have been clinically used as anti-inflammatories both via oral administration and externally for various diseases, the health risks have been extensively assessed. SUL and ACF are also additional candidates with the same affinity range to ZO-1(PDZ1), although their pharmacological investigation against cells is underway. Since SUL has been reported as a potential Wnt signaling inhibitor [23], it may not be an ideal TJ-modulator. ACF is of particular interest because it is partly metabolized into DCF in vivo [30]. Our initial idea that any NSAID can potentially exhibit TJ-opening activity was prompted by our previous finding that DCF and FFA showed an ability to bind accidentally to various PDZ domains [17]. In addition, one of the typical PDZ domains, ZO-1(PDZ1), has been shown to be the key factor in maintaining the integrity of TJs in various cells [15]. We recently proved the idea by demonstrating that baicalin and baicalein show TJ-opening activity, meanwhile these flavonoids also showed weak but specific binding to the pocket of ZO-1(PDZ1) [16]. 

Here, we also demonstrated several technical advantages of solution NMR-based drug screening. We employed a conventional HSQC-based NMR titration experiment, which is sensitive to a weak interaction between the target protein and the ligands of interest. However, interpreting titration experiment results must be done carefully. Therefore, we proposed using a hierarchical cluster analysis. This visualization method is effective to discriminate between specific and non-specific binding. The detailed comparison of this method to other methods, such as “per-residue” plots and PCA, will be reported elsewhere with other application examples.

The pharmacological action of NSAIDs in regulating the epithelial barrier function is still controversial. NSAIDs are either selective or non-selective COX inhibitors that inhibit the biosynthesis of prostaglandins (PGs), such as PGE2. According to the literature, PGE2 may have opposing effects against the TJ integrity of epithelial cells, enhancement, and disruption. Three of the four PGE2 receptor subtypes, EP1, EP2, and EP4, can mediate an increase in cytosolic cAMP or Ca^2+^ concentration, which can decrease TJ integrity via the PKC and PKA pathways, respectively. However, the PGE2/EP3 pathway decreases cAMP, thereby showing TJ enhancement. Therefore, the pharmacological action of PGE2 on the epithelial cells may vary and is depending on the cell types. It has been reported that exogenous and endogenous PGE2 decreases intestinal polyethylene-glycol (PEG) 400 permeability, while INDO reverses the effect [31]. Conversely, PGE2 has been reported to disrupt epithelial TJ integrity in Caco-2 model cell monolayers and to promote intracellular redistribution of OCL and ZO-1 [32]. This process was promoted via EP1 and EP4 and was confirmed by their specific agonists and antagonists. Moreover, two other reports showed disruptive PGE2 action against either the apical junctional complex [33] or the CLD-4-based TJ integrity [34] of Caco-2 cell monolayers. In these contexts, NSAIDs must work as TJ enhancers by inhibiting PGE2 production. However, this assumption seems inconsistent with our results because we observed TJ opening by INDO and DCF. We observed no TJ enhancement by the other two NSAIDs, FLF and IBU. Therefore, there was no TJ enhancement via the action of NSAIDs on PGE2 biosynthesis.

**Figure 7:**
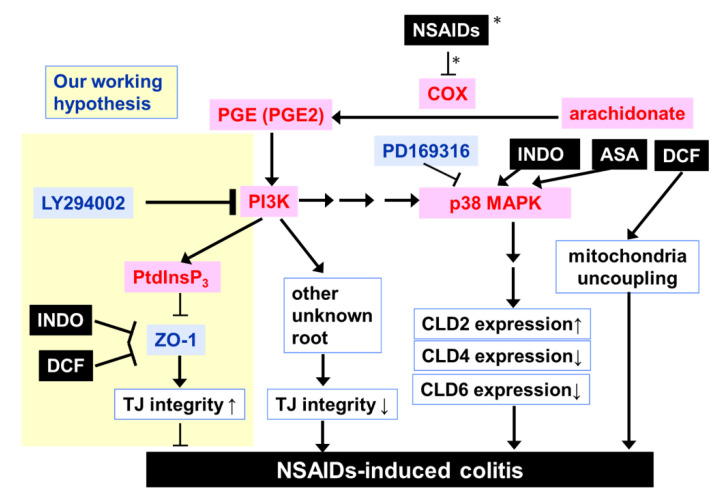
Potential molecular mechanisms of NSAIDs with the downregulation of the integrity of tight junctions. Our current working hypothesis based on the PDZ domain-derived competitive interaction between CLDs and ZO-1 or LNX1 is highlighted in pale yellow. When INDO and DCF interfere with the contact of ZO-1 to CLD-2, LNX1 may promote ubiquitination-dependent internalization of CLD-2 from the membrane; thus, TJ integrity is weakened. Other potential contributions of arachidonate and PGE2 are also illustrated. The substances and enzymes that mediate TJ opening are drawn in red. Only the pathway with an asterisk is that of TJ-enhancement, and the other NSAIDs are decreasing TJ-integrity (see text).

We then focused on the p38 mitogen-activated protein kinase (MAPK) cascade in epithelial TJ regulation, which is potentially activated downstream to PGE2 signaling. It was demonstrated that p38 MAPK activation induces the downregulation of CLD-4 and CLD-8 expression, whereas siRNA against p38 MAPK and PD169316 (an inhibitor of p38) reversed this effect [35]. Two NSAIDs, ASA (aspirin) and INDO, are known to activate p38 MAPK and result in the downregulation of CLD gene expression [36,37,38]. In addition, DCF has been shown to uncouple mitochondrial oxidative phosphorylation and promote TJ disruption via an oxidative stress-dependent mechanism in the primary intestinal cell monolayer system [39]. In this study, we propose an alternative mechanism of NSAID-derived TJ opening by the direct binding of the first PDZ domain of ZO-1, which prevents TJ biogenesis. The signaling cascades discussed here are summarized in Figure 7.

For a long time, the lack of “injection-free” and “needle-free” drug administration pathways for medium molecular-sized drugs has limited their industrial development. Many global pharmaceutical companies have invested in and accelerated the development of orally administrated small drugs. Lipinski’s “rule of five” [40] has been overrated, and the concept of “drug-likeness” has hindered the technical development of medium molecular-sized drugs, including bioactive peptide hormones, cytokines, cyclic peptides, peptide vaccines, and oligonucleotide therapeutics. Our research motivation is to change this situation by developing safe and ready-to-use drug absorption enhancers that are applicable in either the oral or transdermal administration of bioactive peptides, such as insulin. Here, we propose DCF and INDO as useful absorption enhancers in a physiologically relevant concentration.

## 4. Materials and Methods

### 4.1. Materials

ACF, INDO, MFA, and SUL were purchased from TOKYO CHEMICAL INDUSTRY Corporation (Tokyo, Japan). IBU and LOX were purchased from FUJIFILM Wako Pure Chemical Corporation (Osaka, Japan). DCF from Merck-Millipore (Darmstadt, Germany), FLF from Sigma-Aldrich (St. Louis, MO, USA), and ASA from Nacalai Tesque (Kyoto, Japan) were purchased. All NSAIDs used for NMR and cell experiments were dissolved in d_6_-dimethylsulfoxide (DMSO) and DMSO, respectively, except for LOX, dissolved in DMSO and pure water solution. Each stock solution was stored at –20 °C until used. FITC insulin was purchased from Sigma-Aldrich (St. Louis, MO, USA). Rabbit anti-CLD-2 and anti-OCL antibodies were purchased from Sigma-Aldrich. The rabbit anti-ZO-1 antibody was purchased from Invitrogen (Carlsbad, CA, USA). Anti-rabbit immunoglobulin G (IgG) and F(ab’)2 fragment-Cy3 antibody (Sigma-Aldrich) were used for immunofluorescence microscopy. All other reagents were of the highest grade and purity available.

### 4.2. Protein Expression and Purification

Expression and purification of the mouse ZO-1(PDZ1) (residues 18–110) were previously described [41]. In brief, the recombinant glutathione-S-transferase (GST)-tagged form of mouse ZO-1(PDZ1) was uniformly isotopically labeled with ^15^N by harvesting the recombinant *Escherichia coli* BL21(*DE3*) in minimal media with ^15^N-ammonium chloride as the sole nitrogen source. The cells were collected and disrupted, and the cell lysate was applied to GST-accept affinity resin (Nacalai Tesque, Kyoto, Japan) to capture the fusion protein. After on-column digestion by PreScission™ Protease (Cytiva, Tokyo, Japan), ^15^N-labeled ZO-1(PDZ1) was purified by gel filtration chromatography. ZO-1(PDZ1) was dissolved in 5% D_2_O–95% H_2_O containing 20 mM MES buffer (pH 5.9) for further NMR measurements.

### 4.3. NMR Titration Experiments and Data Analysis

To examine the direct binding of the NSAIDs to ZO-1(PDZ1), 2D NMR spectra were recorded at 25 °C by using Avance-III 600 MHz spectrometer (Bruker, Germany) equipped with a cryogenic probe. For ASA, FFA, IBU, INDO, and MFA, 0 and 20 molar equivalent compounds were added to 25 µM ^15^N-labeled ZO-1(PDZ1) and those samples were measured 2D ^1^H-^15^N SOFAST type heteronuclear multiple quantum coherence (SOFAST-HMQC) spectra [42]. For 0 and each indicated equivalent of ACF, LOX, and SUL were added to 0.1 mM ^15^N-labeled ZO-1(PDZ1) and those samples were measured ^1^H-^15^N heteronuclear single quantum coherence (HSQC). Assignments of main-chain NH signals were taken from the entry BMRB 11424 [41]. All the combined chemical shift changes in the ^1^H-^15^N 2D NMR spectra upon ligand titration were calculated in accordance with the Equation (1)

(1) Δδcombined = {Δδ(H1)2 + [Δδ(N15)/6]2}1/2

where Δδ(^1^H) and Δδ(^15^N) are chemical shift changes in amide proton and amide nitrogen, respectively. Visualization of the combined chemical shift changes upon compound binding was performed by the program CCP4mg [43]. The residues with CSPs larger than the threshold were mapped onto the ribbon representation of PDB code 2H3M. Definition of each threshold value followed the method developed by Schumann et al. [24].

Hierarchical clustering of CSP data was performed by R v4.0.2 [44] using the command “*proxy::dist*” in package “proxy” v0.4-24 [45] and “*hclust”*. The dendrograms of CSPs induced by the addition of various NSAIDs were drawn using the command “*plot*”. In this study, we employed “cosine distance” for cluster analysis of CSP data to differentiate the details of various contact residues on ZO-1(PDZ1) upon NSAID binding (see text).

### 4.4. Cell Culture

MDCK II cells were cultured in Dulbecco’s modified Eagle’s medium (DMEM) supplemented with 10% fetal bovine serum (FBS; Biosera, Ringmer, UK) and 1% penicillin/streptomycin (Gibco BRL, Grand Island, NY, USA) at 37 °C with 5% CO_2_. Aliquots of 30 × 10^4^ cells were plated on glass coverslips (Matsunami Glass Industry, Osaka, Japan) in each well of a 6-well, 35-mm plate (Corning Japan, Tokyo, Japan) and were kept for 24 h after plated. For NSAIDs treatment, the culture medium was changed to the medium containing 100 μM of DCF, FFA, IBU, and INDO. After 48 h of exposure to the compounds, the cells were subjected to immunofluorescence. Cells exposed to 0.1% DMSO were used as a control.

### 4.5. Immunofluorescence Microscopy

The cells were fixed with cold 1 × phosphate-buffered saline containing 4% paraformaldehyde. The fixed cells were then incubated with primary antibodies overnight at 4 °C, followed by 1 h incubation at room temperature with the corresponding secondary antibodies. Fluorescence microscopy equipped with a color CCD camera (IX-71 and DP-70, respectively; Olympus, Tokyo, Japan; scale bar: 20 μm) was used to obtain the immunofluorescence microscope images.

### 4.6. Transepithelial Resistance (TEER)

Caco-2 cells were cultured to a tight monolayer on the upper transwell filter of a Millicell-24 cell culture plate (Merck-Millipore, Darmstadt, Germany) and maintained for approximately 14 days until stable transepithelial electrical resistance (TEER) was achieved. The standard culture medium (Minimum Essential Medium Eagle, Sigma, supplemented with 10% FBS (Biosera, Kansas City, MO, USA), 1% penicillin/streptomycin (Gibco), and 1% MEM-NEAA (Gibco) was used and changed every 2 days. An epithelial volt-ohm meter (Millicell^®^ ERS-2, Merck-Millipore) was used to measure the TEER of the cell monolayer. The cells with stable TEER were then exposed to 300 μM of NSAIDS, where 0.3% DMSO was a diluent control.

### 4.7. Paracellular Permeation Assay

The Caco-2 cell monolayers on the upper transwell filter of a Millicell-24 cell culture plate were prepared similarly to the TEER assay and confirmed that the TJ was well maturated based on their constant TEER values (data not shown). Subsequently, the cells on the filter were incubated with above standard medium (400 µl) supplemented with 300 μM of DCF or INDO or 0.3% DMSO and 0.44 μM FITC-insulin as the paracellular flux tracer. Subsequently, 800 μL of the standard culture medium was added to the basal side chamber. At given times, each 100 μL of the sample from the basal side was taken and placed in a black 96-well plate (Perkin-Elmer, Norwalk, CT, USA). The fluorescence was measured with an EnSpire plate reader (Perkin-Elmer) excited at 488 nm and emitted at 509 nm. 

## 5. Conclusions

Six out of nine NSAIDs—DCF, LOX, ACF, SUL, FFA, and INDO, all of which harbored a carboxylate group—were found to bind the canonical claudin-binding pocket of ZO-1(PDZ1) weakly but specifically, assessed using solution NMR techniques. To analyze a weak but specific molecular interaction of NSAIDs, we employed a novel hierarchical clustering analysis method for the traditional NMR titration experiments. We examined four NSAIDs for their TJ modulation activity against MDCK II and Caco-2 cells. DCF, FFA, and INDO exhibited activity to decrease the subcellular accumulation of CLD-2 but not OCL and ZO-1 in the intercellular TJ compartment. FFA showed additional activity of cell area expansion. DCF and INDO exhibited the activity of enhancing the efflux of fluorescent analog of insulin beyond the monolayer of Caco-2 cells, whose molecular weight is approximately 6000. We propose DCF and INDO as potential candidates to develop drug absorption enhancers that can be applicable for oral insulin administration.

## Data Availability

The datasets generated during and/or analyzed during the current study are available from the corresponding author on request.
